# Transparent Cellulose/Technical Lignin Composite Films for Advanced Packaging

**DOI:** 10.3390/polym11091455

**Published:** 2019-09-05

**Authors:** Yujie Guo, Dong Tian, Fei Shen, Gang Yang, Lulu Long, Jinsong He, Chun Song, Jing Zhang, Ying Zhu, Churui Huang, Shihuai Deng

**Affiliations:** 1 Institute of Ecological and Environmental Sciences, Sichuan Agricultural University, Chengdu 611130, Sichuan, China; 2 State Key Laboratory of Polymer Materials Engineering, Polymer Research Institute of Sichuan University, Chengdu 610065, Sichuan, China

**Keywords:** lignin, UV-shielding, antioxidant, cellulose film, pretreatment

## Abstract

Although recent work has shown natural lignin products are promising to fabricate various polymer based functional composites, high-value applications were challenged by their structural complexity and inhomogeneity. This work specially assessed the potential of four technical lignins for cellulose based functional films production. These four technical lignins were obtained by emerging pretreatment systems, i.e., lactic acid-betaine deep eutectic solvent (DES), ethanol organosolv, soda/anthraquinone (Soda/AQ) and the sodium salicylate hydrotrope, and their phenolic substructures were comparatively identified by prevalent ^31^P NMR technique. The influence of lignin chemical structure on the antioxidant potential and UV-shielding performance of the prepared cellulose/technical lignin composite films were assessed. Results showed severe organosolv and soda/AQ pretreatment produced technical lignins with higher total phenolic hydroxyl groups (3.37 and 3.23 mmol g-1 respectively), which also exhibited higher antioxidant activities. The composite films could effectively block the ultraviolet lights especially for UVB region (ultraviolet B, 280–315 nm) at only 5 wt.% lignin content. The contribution of lignin phenolic substructures to both antioxidant activity and UV-shielding property from high to low was syringyl > guaiacyl > *p*-hydroxyphenyl phenolic hydroxyl groups. This work provided some useful information that could facilitate upstream lignin extraction or downstream value-added applications.

## 1. Introduction

Traditional packaging materials such as polyethylene and polypropylene are mainly produced from non-renewable petroleum resources which has been causing serious environmental concerns due to their ultimate disposal and non-biodegradability [1]. A promising technical strategy to deal with this challenge is to use natural polymers to replace these synthetic polymers for packaging materials production. Cellulose is the most abundant and renewable biopolymer on the earth. It holds great promise for a wide range of types of materials’ fabrication such as cellulose nanofibers, films, hydrogels and aerogels for downstream applications due to their abundance in nature, excellent mechanical properties and biodegradability [2,3,4]. When cellulose is subjected to a dissolution-casting process, flexible transparent films with excellent barrier properties could be obtained, which are comparable to those of prevalent plastic packaging materials [5]. Therefore, these transparent cellulose films are promising alternatives to synthetic plastics for next-generation packaging materials production.

To enhance the value of packaging materials, additional functionality including antioxidant, antimicrobial and UV-shielding property has been usually introduced by incorporating various functional additives into the polymer matrix during the film production process. Although prevalent metal oxides such as titanium dioxide and zinc oxide are promising UV-absorber additives, potential environmental hazards are accompanied when they are disposed after use [6]. In addition, these metal oxides could also catalyze the polymer matrix degradation due to their intrinsic photocatalytic activity [7,8,9]. Recent research progress has shown that some natural biopolymers that contain phenolic hydroxyl groups could be used as both antioxidants and UV-shielding additives to replace the above metal oxides. Unlike metal oxides that block UV lights though a “reflection” manner, these biopolymers are supposed to block UV lights through absorbing their photon energy, which was further converted to heat with the corresponding functional chromophores [10]. Considering that lignin biopolymer is abundant in phenolic hydroxyl groups and compatible with cellulose, it might be a promising functional additive to endow both antioxidant and UV-shielding functionality to cellulose film towards an advanced composite packaging material.

However, lignin in nature is a highly heterogeneous biopolymer. It is mainly composed of three phenylpropane units (mono-lignols), namely, syringyl (S, sinapyl alcohol), guaiacyl (G, conniferyl alcohol), and p-hydroxyphenyl (H, p-coumaryl alcohol) [11,12]. The complex and heterogeneous chemical structure challenges its downstream applications especially when it is used as a functional bio-additive in which the content and location of phenolic hydroxyl groups play a determinant role. It has been acknowledged that apart from biomass species, the selected pretreatment route that is used to extract lignin from lignocellulosic biomass in a typical biorefinery is also influential in lignin chemical structure [13]. For example, acid based pretreatment tends to easily cleave β−O−4′ linkages in lignin but cause severe condensation reactions among aromatics while ethanol based organosolv pretreatment tends to produce lignin with low molecular weight and rather high chemical reactivity [14]. Our previous work has shown that four emerging lignocellulose pretreatment methods, i.e., lactic acid-betaine deep eutectic solvent (DES), ethanol organosolv, green liquor (soda/anthraquinone, soda/AQ) and the sodium salicylate hydrotrope could facilely ease cellulose hydrolysis from steam pretreated poplar wood, while producing a technical lignin faction with varied yields. Incorporating these lignin fractions into cellulose matrix to enhance its antioxidant and UV-shielding performance not only shows new opportunities to produce a cellulose based advanced packaging material, but also adds an additional product stream for the current lignocellulose biorefineries [15].

In the work reported here, the technical potential of preparation of transparent cellulose/technical lignin composite films using prevalent solution-casting method was assessed. Four emerging technical lignins were comparatively assessed for their potential to enhance the antioxidant and UV-shielding properties of the resulting composite films. Since lignin phenolic hydroxyl groups were the corresponding functional groups, the influence of their locations and contents on the antioxidant and UV-shielding performance was particularly investigated after they were analyzed by ^31^P NMR technique. To give a better assessment, commercial Kraft lignin was introduced in all the experiments. This work showed a promising way to valorize lignin byproduct through fabricating cellulose/technical lignin composite films for advanced packaging, and also provided useful information for lignin downstream application when it was used as an antioxidant and UV-shielding bio-additive.

## 2. Materials and Methods

### 2.1. Materials

Steam pretreated poplar wood was kindly obtained from Forest Products Biotechnology/Bioenergy Group, Department of Wood Science (University of British Columbia, Vancouver, BC, Canada). Diphenyl-1-picrylhydrazyl (DPPH), 6-hydroxy-2,5,7,8-tetramethylchroman-2-carboxylic acid (Trolox), dimethyl sulfoxide (DMSO), cyclohexanol, anhydrous pyridine, deuterated chloroform, chromium(III) acetylacetonate, 2-chloro-4,4,5,5-tetramethyl-1,3,2-dioxaphospholane (TMDP, Product No. 447536), betain, sodium salicylate, anthraquinone, commercial Kraft lignin (Product No. 370959) and microcrystalline cellulose (Avicel^®^ PH-101) were purchased from Sigma-Aldrich. Other common chemicals and organic solvents with analytical grade were purchased from Kelong Chemical Regent Co., Ltd. (Chengdu, China).

### 2.2. Lignin Extraction and Characterization

Lignin extraction was conducted using the two-stage pretreatments, i.e., steam pretreatment followed by DES, soda/AQ, hydrotrope and organosolv according to our previous work [16]. DES was first synthesized by stirring lactic acid and betain at a molar ratio of 2.5:1 at 80 °C until a homogeneous colorless liquid was formed [17]. Then resulting DES was applied for lignin extraction from the steam pretreated poplar substrate under atmosphere pressure. About 10 g steam pretreated poplar biomass (based on dry weight) and 200 g DES was transferred to a flat bottom glass flask and heated at 130 °C for 3 h on a hot plate with continuous stirring. To remove DES and lignin traces, the resulting cellulose pulp was washed by 30 mL of acetone-water mixture (1:1, *v*/*v*) twice and then hot water twice [18]. The washes and the darkly-colored solution containing lignin and DES were combined together, then placed in fume hood for two days in order to precipitate the dissolved lignin by evaporating of acetone. Lignin extractions with organosolv [19], soda/AQ [20,21] and hydrotrope [22,23] were conducted at their optimum conditions respectively according to previous study. Briefly, dried 100 g steam pretreated poplar wood was cooked at 170 °C for 1 h (liquid to solid ratio, 7:1). The solvent composition was ethanol/water, 50/50 by weight, 1% H_2_SO_4_ for Organosolv extraction, sodium carbonate in water (14% active alkali), 0.1% anthraquinone (AQ) for Soda/AQ extraction and 30% sodium salicylate in water with 0.17% formic acid catalyst for hydrotrope extraction respectively. After the pretreatment, the vessels were cooled to room temperature in the water bath. The liquid fractions were then collected through vacuum filtration. Lignin precipitation was conducted by adding hot water 10 times to the filtrate fraction for Organosolv and Hydrotrope process, or adjusting the pH to 2.0 with concentrated H_2_SO_4_ for Soda/AQ process.

The four lignin precipitates were finally collected on nylon membrane, washed thoroughly with water to pH neutral, dried in the vacuum oven and stored in a desiccator for further characterization use.

Quantitative ^31^P NMR spectra were used to analyze various hydroxyl group contents according to previous work [24,25]. Approximately 20 mg of various lignin (dry sample) was completely dissolved in 500 μL of freshly prepared mixture of anhydrous pyridine and deuterated chloroform (1.6:1, *v*/*v*). Then 100 μL of cyclohexanol (10.85 mg mL^−1^ in the above pyridine and deuterated chloroform mixture) was added as an internal standard and 100 μL of chromium(III) acetylacetonate solution (5 mg mL^−1^ in the pyridine and deuterated chloroform mixture) was added as the relaxation reagent. Afterward, 100 μL phosphitylating reagent (TMDP) was rapidly added. The prepared sample mixture was further transferred to a 5 mm NMR tube for spectra acquisition (AV II 600, Bruker) [26,27]. The chemical shift of aliphatic -OH, internal standard, Syringyl -OH, Guaiacyl -OH, p–hydroxyphenyl -OH and -COOH was identified at 145.5–150.0 ppm, 144.7–145.5 ppm, 140.5–144.5 ppm, 138.6–140.4 ppm, 137.3–138.6 ppm and 133.7–136.0 ppm respectively.

### 2.3. Preparation of Cellulose/Technical Lignin Composite Films

An 8% (*w*/*w*) DMAc/ LiCl solvent system was prepared according to previous work [28]. Avicel^®^ PH-101 cellulose was activated by methanol and DMAc from the previous procedure before dissolution. The activated Avicel^®^ PH-101 cellulose was added to the above 8% (*w*/*w*) DMAc/LiCl solvent system with string until a homogeneous transparent solution was obtained.

Next, 0.2 g of each lignin sample was added into the above cellulose solution to get a final lignin content of 5 wt.% (cellulose basis), respectively. The resultant mixture was casted on a glass plate and kept at ambient conditions overnight to form a gel-like film. To remove DMAc and LiCl traces, the gel-like film was washed thoroughly with distilled water for 24 h. Then the obtained cellulose/lignin composite film was dried in a vacuum oven.

### 2.4. Antioxidant Activity and UV-Shielding Measurement

The free radical scavenging ability of lignin was evaluated according to a published method reported by Delgado-Andrade et al. with a few modifications [29]. Then, 7.4 mg DPPH was dissolved in 100 mL methanol to obtain an absorbance of 1.8 at 520 nm. Then 200 µL lignin solution (0.1–1.0 mg mL^−1^ in DMSO) was mixed with 1 mL of 74 mg L^−1^ DPPH in methanol and incubated for 1 h at 30 °C. After the reaction, its absorption at 520 nm was immediately measured [30,31]. Trolox solutions with different concentrations (0.1–1 mmol L^−1^) in DMSO were used as the standard. The results were calculated and expressed as µmol equivalents to Trolox per g of each lignin sample.

The UV-shielding performance of the prepared composite films was tested on a UV-Visible spectrophotometer (U-2910, HITACHI, Tokyo, Japan). The spectra was obtained at a scanning step of 2 nm from 200 to 800 nm [32].

## 3. Results and Discussion

**Scheme 1:**
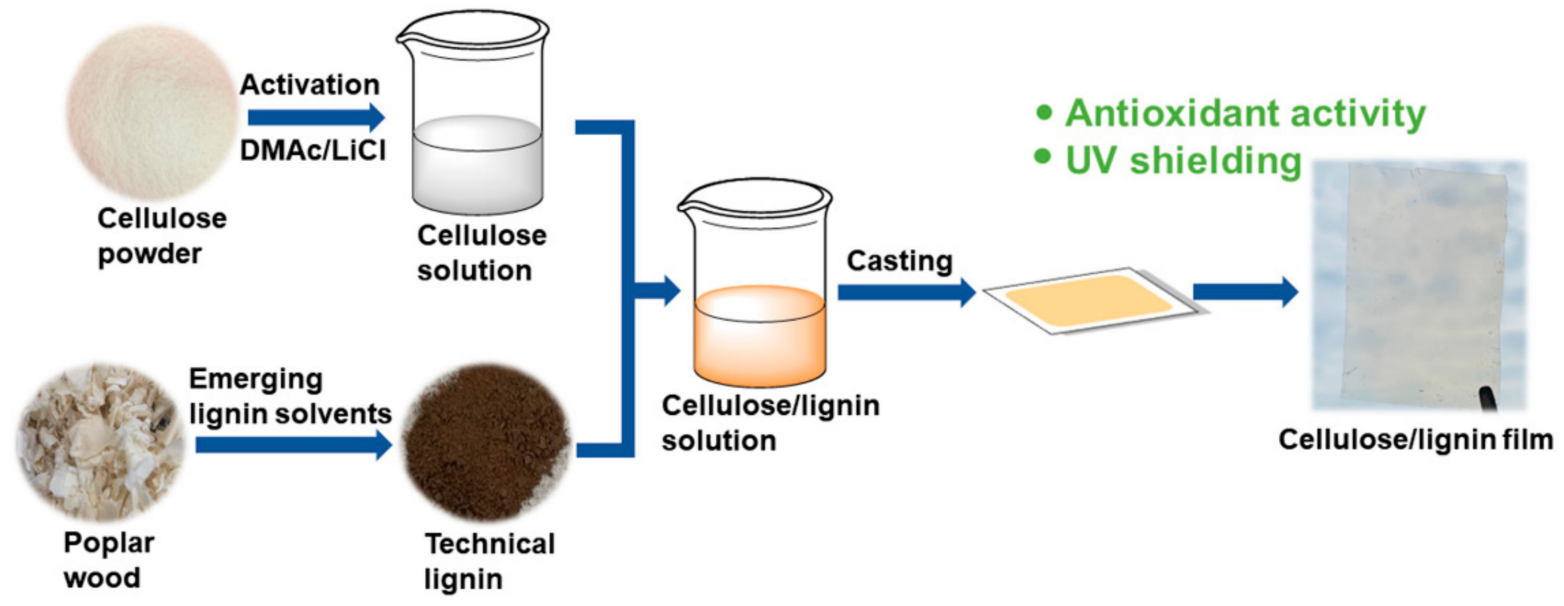
Procedure to fabricate transparent cellulose/technical lignin composite films with enhanced antioxidant and UV-shielding property. Technical lignins were extracted by deep eutectic solvent, ethanol organosolv, soda/anthraquinone and a hydrotrope respectively at their optimal conditions.

The detailed procedure to fabricate the transparent cellulose/technical lignin composite films was shown in Scheme 1. DMAc/LiCl was selected as the processing solvent for demonstrating purpose since it has good solubility for both cellulose and lignin. Four technical lignins, i.e., DES, organosolv, soda/AQ and hydrotrope lignin were extracted and added respectively into cellulose solution in DMAc/LiCl with 5 wt.% (based on the weight of dry cellulose). Then the mixed cellulose/lignin solution was casted and solvent-exchanged with water to produce the composite films. It was proposed that the phenolic hydroxyl groups in these technical lignins could easily scavenge active free radicals through an electron transfer process, therefore endowing the composite films with enhanced antioxidant property. The phenolic hydroxyl groups could also shield UV lights through absorbing their photon energy and then converting it to heat, which was further gradually released into the environment without causing the photo-catalytic degradation of cellulose matrix in comparison to metal oxides.

**Figure 1:**
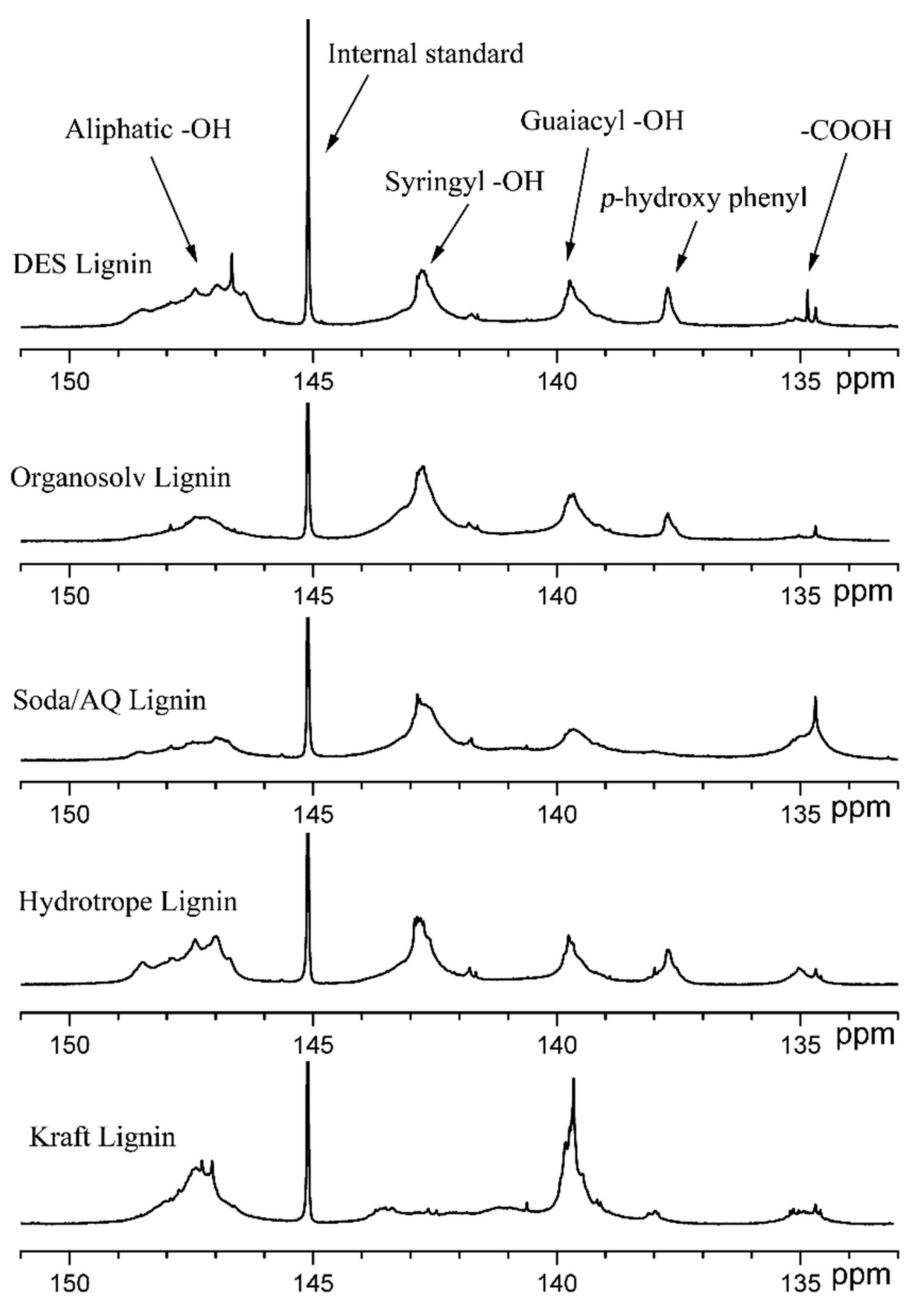
Quantitative ^31^P NMR spectra of these four technical lignins and Kraft lignin tagged TMDP phosphorous reagent using cyclohexanol as internal standard.

^31^P NMR spectroscopy is a useful technique to quantitatively analyze various lignin hydroxyl groups [26]. The basic principle is that both the internal standard and the five testing lignin sample are completely soluble and phosphitylated with the phosphorous reagent in the pyridine/deuterated chloroform solvent system, then the phosphorus-labeled hydroxyl groups that belong to lignin including phenolic, aliphatic, and carboxylic hydroxyls could be facilely quantified by ^31^P NMR spectroscopy [26]. The quantitative ^31^P NMR spectra of these four technical lignins and Kraft lignin were shown in Figure 1 and the calculated results were presented in Table 1 accordingly. It was apparent that these four technical lignins exhibiting dominant S and G phenolic hydroxyl groups were the HGS (hydroxy−guaiacyl−syringyl)-type hardwood lignin whereas Kraft lignin was the G-type softwood lignin. The structural complexity and heterogeneity of these lignins were evidenced by the multiple peaks in their ^31^P NMR spectra. It was shown these four extraction solvent systems varied in their abilities to alter lignin chemical structures, thus, their ^31^P NMR spectra were quite different from each other (Figure 1).

In a typical lignin extraction process, lignin fragmentation through β−O−4′ linkages cleavage and lignin condensation among aromatics are two dominant reactions. Lignin fragmentation generates more phenolic units while lignin condensation generates stable carbon–carbon bonds between aromatics [33]. When their total phenolic hydroxyl groups contents were compared (Table 1), it was apparent that mild DES extraction caused lower extent of lignin fragmentation, thus, the resulting DES lignin exhibited a low content of total phenolic hydroxyl groups (2.44 mmol g^−1^). The other three technical lignins exhibited considerably high contents of total phenolic groups (3.06~3.37 mmol g^−1^), which was higher or comparable to Kraft lignin (3.18 mmol g^−1^). In addition to higher extent lignin fragmentation and condensation, severe ethanol organosolv and soda/AQ solvent system also significantly dehydrated aliphatic hydroxyl groups (1.04 and 1.17 mmol g^−1^ respectively). While soda/AQ process tended to oxidize lignin aromatics through a ring-opening reaction, the resulting technical lignin exhibited much higher content of carboxylic acid hydroxyl groups (0.87 mmol g^−1^). It was also apparent that the softwood Kraft lignin exhibited much higher content of G phenolic hydroxyl groups (2.11 mmol g^−1^) in comparison to these four hardwood technical lignins (0.90~1.31 mmol g^−1^). From the analysis above, it was shown that the contents and types of phenolic hydroxyl groups in these four technical lignins varied among each other despite the fact that the same starting poplar wood substrate was used. The extraction route selected was highly determinable in lignin chemical structures.

**Figure 2:**
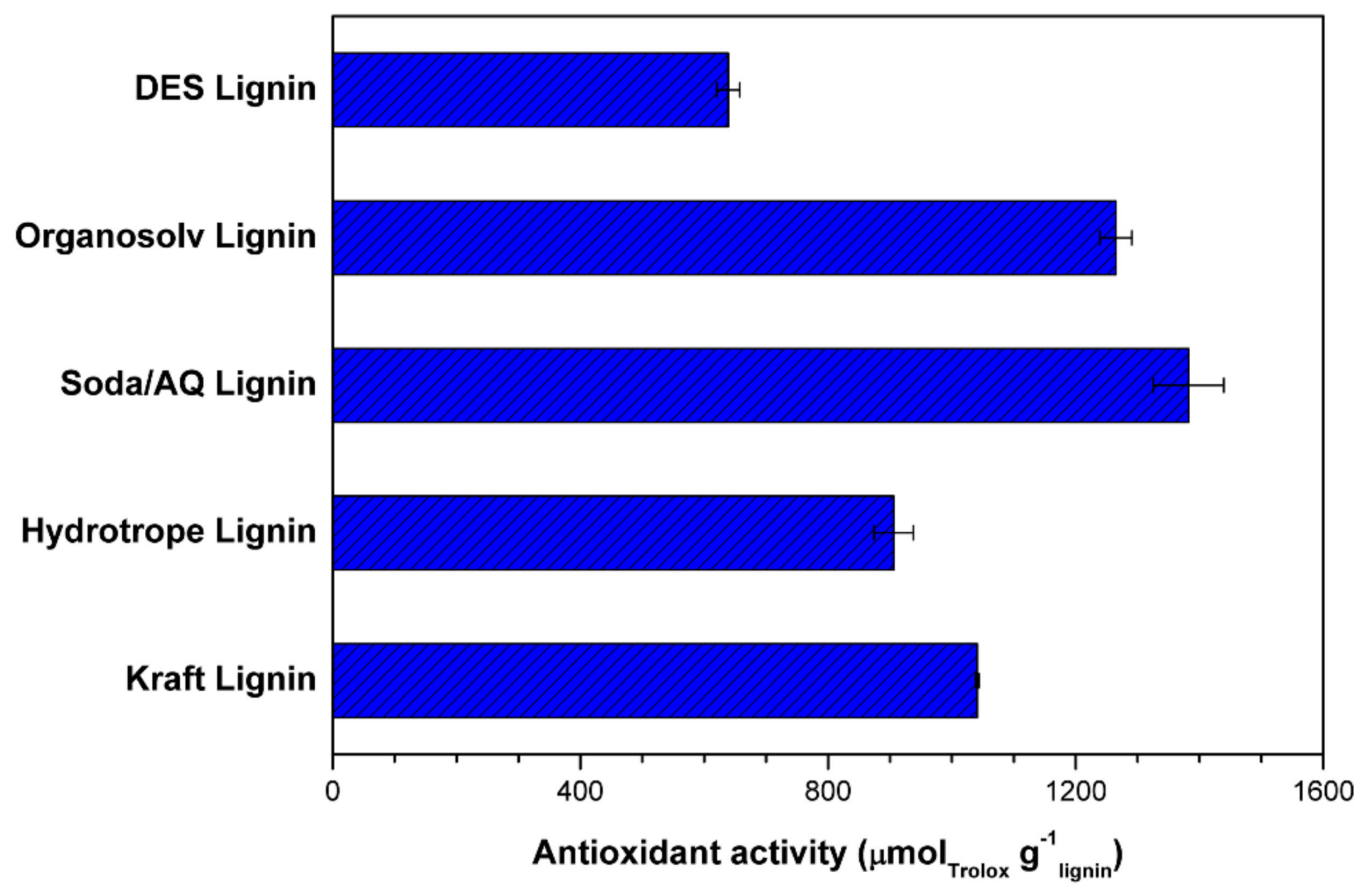
Antioxidant activity of these four technical lignins and Kraft lignin determined by Trolox equivalent antioxidant capacity (TEAC) assay.

The antioxidant properties of those lignins were further investigated using facile Trolox equivalent antioxidant capacity (TEAC) assessment. The TEAC method employed stable DPPH as the testing radicals and Trolox as the internal standard respectively according to previous reports (Figure 2) [24,29]. The antioxidant activity of these four technical lignins was ranging from 683 to 1382 μmol_Trolox_ g^−1^_lignin_, which was comparable to that of natural phenolic compounds [29,34]. Among these four technical lignins, the antioxidant activity of soda/AQ and organosolv lignin was similar to that of tea polyphenols [35]. The antioxidant activity of DES lignin was still higher than that of some common fruit extracts such as banana and watermelon extract [36]. These results indicated the high feasibility of those technical lignins for the application antioxidant purpose. It was interesting that although organosolv, soda/AQ and Kraft lignin exhibited similar total phenolic hydroxyls groups, their antioxidant activities were quite varied, indicating that the activity of each type of phenolic hydroxyls was dependent. It appeared that syringyl phenolic hydroxyl groups were more responsible for radical-scavenging. When soda/AQ and organosolv lignin were compared, it seemed that the additional carboxylic acid hydroxyl groups in soda/AQ lignin significantly enhanced the overall availability of their phenolic hydroxyls groups, corresponding to an increased antioxidant activity of 1382 μmol_Trolox_ g^−1^_lignin_ for soda/AQ lignin.

**Figure 3:**
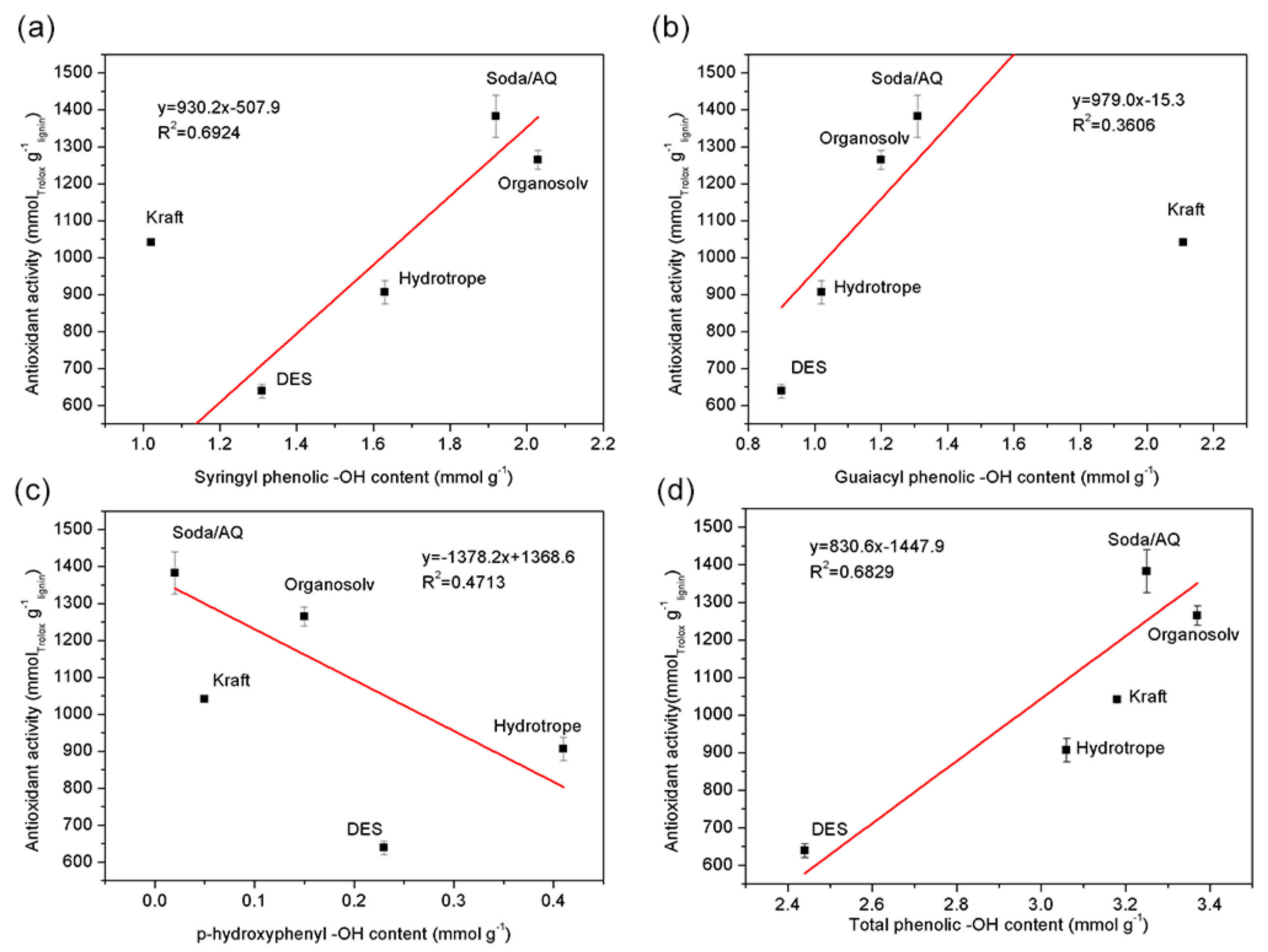
Relationship among antioxidant activity and the content of (**a**) syringyl phenolic hydroxyl groups, (**b**) guaiacyl phenolic hydroxyl groups, (**c**) p-hydroxyphenyl phenolic hydroxyl groups and (**d**) total phenolic hydroxyl groups of those five lignins.

Specifically, the antioxidant activity of those five lignins was plotted against their syringyl, guaiacyl, p-hydroxyphenyl and total phenolic hydroxyl groups content respectively to understand which type of phenolic hydroxyl groups was more dominant (Figure 3). It was shown the antioxidant activity was more positively correlated with the content of syringyl phenolic hydroxyl groups (R^2^ = 0.6924), followed by guaiacyl phenolic hydroxyl groups among those five lignins [37]. Furthermore, p-hydroxyphenyl phenolic hydroxyl groups showed limited contribution to antioxidant activity due to their rather low content, especially for soda/AQ and Kraft lignin. The overall higher antioxidant activities of those lignins were roughly correlated with their higher total phenolic hydroxyl groups (Figure 3), which was in line with previously reported work that lignin with lower molecular weights tended to show higher antioxidant activity [38]. Although the antioxidation potential of various lignins have been extensively explored recently, results reported here suggest that lignin with higher syringyl units is more promising.

**Figure 4:**
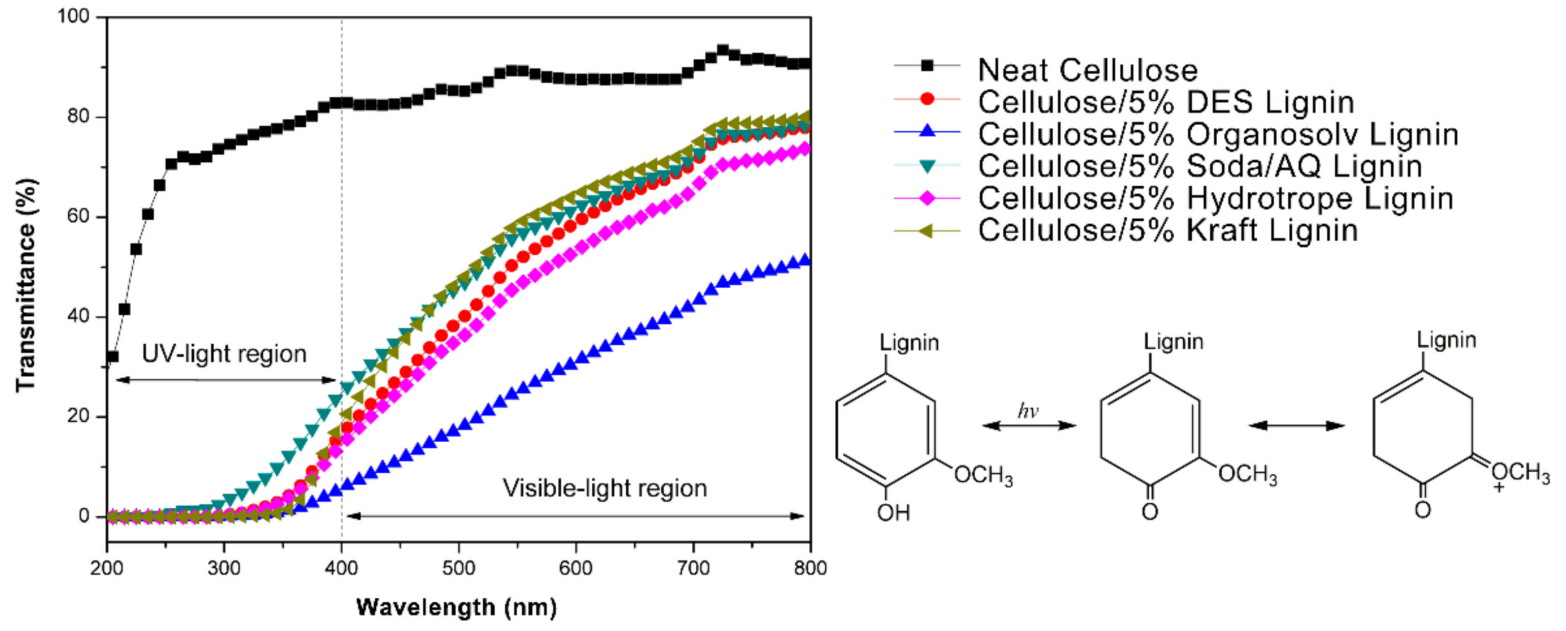
UV–Vis light transmittance spectra of cellulose/technical lignin composite films and possible mechanism of how methoxy groups in syringyl and guaiacyl phenolic hydroxyl groups enhanced their UV-shielding performance.

The above results showed the high promise of incorporating these technical lignins into cellulose matrix to enhance their antioxidant performance. To further assess the influence of their phenolic substructures on the UV-shielding performance of the fabricated cellulose/technical lignin composite films, UV–Visible light scanning was subsequently conducted at the wavelength from 200 to 800 nm. It was shown that all those five cellulose/lignin composite films could efficiently block the ultraviolet lights especially for UVB region (ultraviolet B, 280–315 nm) at 5 wt.% lignin content, while the neat cellulose film was almost transparent for all the testing ultraviolet lights (Figure 4). Although the excellent UV-shielding performance was achieved at the compromise of their transparency at visible-light region, considerable transparency was still obtained (72~79% at 800 nm) except for cellulose/ 5% organosolv lignin composite film (51% at 800 nm).

**Figure 5:**
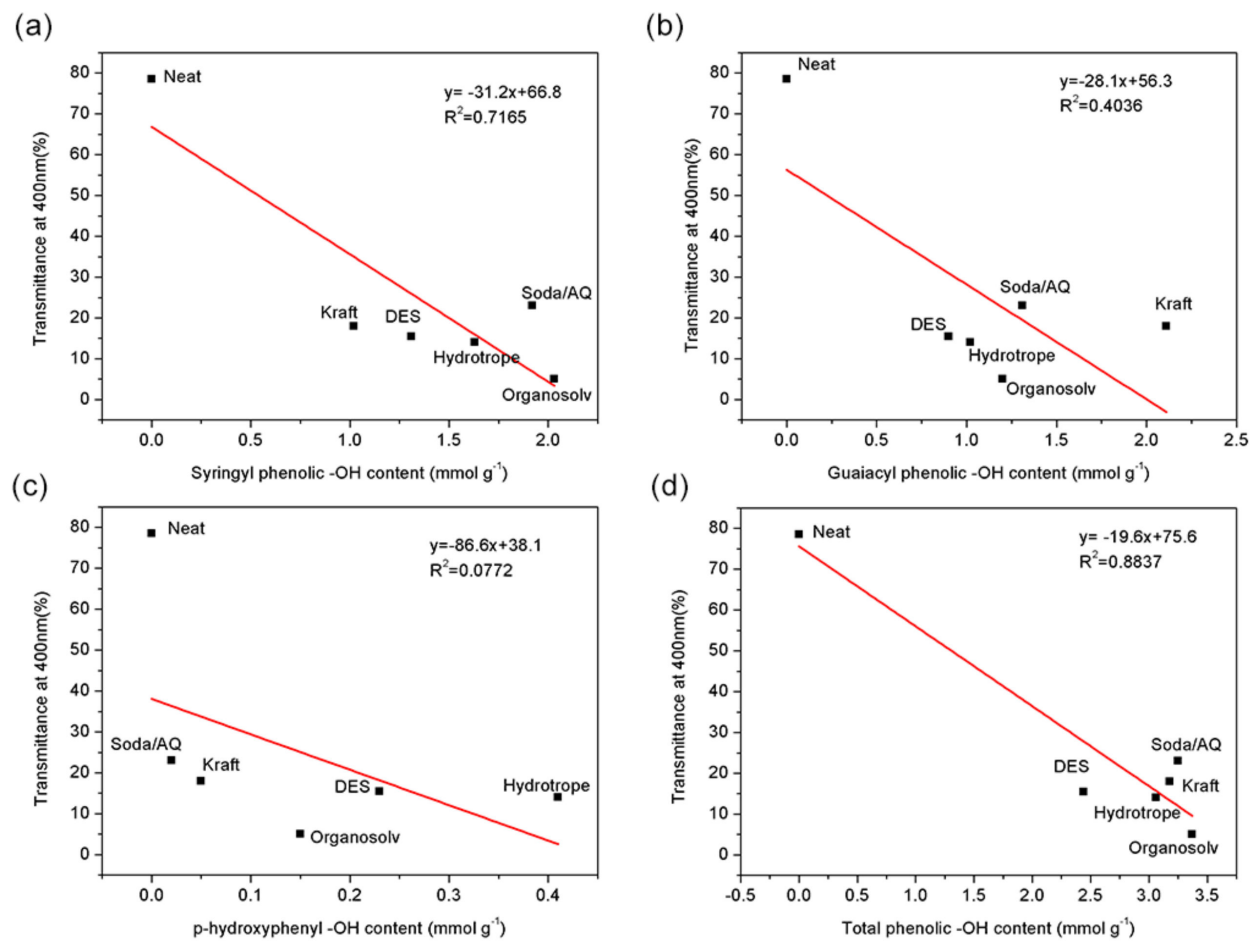
Relationship among transmittance at 400 nm of composite films and the content of (**a**) syringyl phenolic hydroxyl groups, (**b**) guaiacyl phenolic hydroxyl groups, (**c**) p-hydroxyphenyl phenolic hydroxyl groups and (**d**) total phenolic hydroxyl groups of those five lignins.

Similar to the results of the above antioxidant activity assessment, when the transmittance values at 400 nm of those composite films were plotted against the contents of syringyl, guaiacyl, p-hydroxyphenyl and total phenolic hydroxyl groups of the corresponding lignins (Figure 5), the higher contents of total phenolic hydroxyl groups correlated well with their lower transmittance values at 400 nm (R^2^ = 0.8837). It was also apparent that syringyl phenolic hydroxyl groups were more successful in ultraviolet lights blocking (R^2^ = 0.7165) than guaiacyl phenolic hydroxyl groups (R^2^ = 0.4036). The possible reason was that methoxy groups locating on lignin aromatic rings disrupted the electrons distribution which allowed the phenolic hydroxyl groups to stay at active state. When they were exposed to ultraviolet lights [39], lower band-gap energy was required to change the chromophores to excited state, indicating that more ultraviolet lights with wider wavelength range could be blocked (Figure 4).

From the analysis above, it was shown that the contribution of lignin phenolic substructures to both antioxidant activity and UV-shielding property from high to low was syringyl > guaiacyl > p-hydroxyphenyl phenolic hydroxyl groups. Organosolv and soda/AQ lignin that exhibited considerably higher contents of syringyl phenolic hydroxyl groups could potentially endow cellulose/lignin composite films with both considerable antioxidant and UV-shielding performance. These technical lignins were more promising for downstream applications when they were used as bi-functional bio-additives for advanced packaging materials purpose such as active drugs storage.

## 4. Conclusions

This work showed that poplar wood lignin chemical structures are highly determined by the upstream pretreatment approach selected. Both the contents and locations of lignin phenolic substructures are influential in the antioxidant and UV-shielding performance of the prepared cellulose/technical lignin composite films. Syringyl phenolic units containing additional methoxy groups were more influential in both antioxidant and UV-shielding properties. Aggressive organosolv and soda/AQ pretreatments that could effectively solubilize and fragment biomass lignin are preferred if the resulting technical lignin is used as antioxidant and UV-shielding functional bio-additive. The prepared composite films are promising for advance packaging for active drugs, long-time food storage and biodegradable packaging, etc. This work also shows a promising way to valorize lignin that is usually disposed as a by-product in a lignocellulose biorefinery.

## Figures and Tables

**Table 1:** Contents and locations of various hydroxyl groups in these five lignins quantified by ^31^P NMR technique.

–OH Content (mmol g^−1^)	DES Lignin	Organosolv Lignin	Soda/AQ Lignin	Hydrotrope Lignin	Kraft Lignin
Aliphatic -OH	2.40	1.04	1.17	2.00	1.98
Syringyl phenolic -OH	1.31	2.03	1.92	1.63	1.02
Guaiacyl phenolic -OH	0.90	1.20	1.31	1.02	2.11
p-hydroxyphenyl -OH	0.23	0.15	0.02	0.41	0.05
Carboxylic acid -OH	0.18	0.09	0.87	0.32	0.37
Total phenolic -OH	2.44	3.37	3.25	3.06	3.18
